# Effect of SGLT2-Inhibitors on Polygraphic Parameters in Elderly Patients Affected by Heart Failure, Type 2 Diabetes Mellitus, and Sleep Apnea

**DOI:** 10.3390/biomedicines12050937

**Published:** 2024-04-23

**Authors:** Giuseppe Armentaro, Corrado Pelaia, Valentino Condoleo, Giandomenico Severini, Giulia Crudo, Mario De Marco, Carlo Alberto Pastura, Valeria Tallarico, Rita Pezzella, Domenico Aiello, Sofia Miceli, Raffaele Maio, Gianluigi Savarese, Giuseppe M. C. Rosano, Angela Sciacqua

**Affiliations:** 1 Department of Medical and Surgical Sciences, University “Magna Græcia” of Catanzaro, Campus Universitario “S. Venuta”, Viale Europa—Località Germaneto, 88100 Catanzaro, Italy; giuseppearmentaro91@gmail.com (G.A.); pelaia.corrado@gmail.com (C.P.); condoleovalentino@gmail.com (V.C.); giandomenicoseverini@gmail.com (G.S.); crudogiulia@gmail.com (G.C.); mariodemarco@live.it (M.D.M.); carloalbertopastura@gmail.com (C.A.P.); sofy.miceli@libero.it (S.M.); raf_maio@yahoo.it (R.M.); 2 Pediatric Division, AOU Renato Dulbecco, 88100 Catanzaro, Italy; valeria.tallarico@libero.it; 3 Department of Translational Medical Sciences, Federico II University of Naples, 80131 Naples, Italy; ritapezze@gmail.com; 4 Department of Health Sciences, University “Magna Græcia” of Catanzaro, 88100 Catanzaro, Italy; d.aiello@unicz.it; 5 Division of Cardiology, Department of Medicine, Karolinska Institutet, 171 77 Stockholm, Sweden; gianluigi.savarese@ki.se; 6 Department of Human Sciences and Promotion of Quality of Life, Chair of Pharmacology, San Raffaele University of Rome, 00166 Rome, Italy; giuseppe.rosano@gmail.com; 7 Cardiology, San Raffaele Cassino Hospital, 03043 Cassino, Italy

**Keywords:** elderly, heart failure, sleep apnea, SGLT2i, T2DM

## Abstract

Obstructive sleep apneas (OSAs) and central sleep apneas (CSAs) are the most common comorbidities in Heart Failure (HF) that are strongly associated with all-cause mortality. Several therapeutic approaches have been used to treat CSA and OSA, but none have been shown to significantly improve HF prognosis. Our study evaluated the effects of a 3-months treatment with sodium-glucose cotransporter type 2 inhibitor (SGLT2i) on polygraphic parameters in patients with sleep apnea (SA) and HF, across the spectrum of ejection fraction, not treated with continuous positive air pressure (CPAP). A group of 514 consecutive elderly outpatients with HF, type 2 diabetes mellitus (T2DM) and SA, eligible for treatment with SGLT2i, were included in the investigation before starting any CPAP therapy. The two groups were compared with the *t*-test and Mann–Whitney test for unpaired data when appropriate. Then, a simple logistic regression model was built using 50% reduction in AHI as the dependent variable and other variables as covariates. A multivariate stepwise logistic regression model was constructed using the variables that linked with the dependent variable to calculate the odds ratio (OR) for the independent predictors associated with the reduction of 50% in AHI. The treated group experienced significant improvements in polygraphic parameters between baseline values and follow-up with reduction in AHI (28.4 ± 12.9 e/h vs. 15.2 ± 6.5 e/h; *p* < 0.0001), ODI (15.4 ± 3.3 e/h vs. 11.1 ± 2.6 e/h; *p* < 0.0001), and TC90 (14.1 ± 4.2% vs. 8.2 ± 2.0%; *p* < 0.0001), while mean SpO_2_ improved (91. 3 ± 2.3 vs. 93.8 ± 2.5); *p* < 0.0001. These benefits were not seen in the untreated population. The use of SGLT2i in patients suffering from HF and mixed-type SA not on CPAP therapy significantly contributes to improving polygraphic parameters.

## 1. Introduction

Heart Failure (HF) is a clinical syndrome that is widely prevalent and has significant global social and economic repercussions, with a prevalence of more than 10% in elderly persons in Western nations [1,2]. Despite recent advancements in the pharmaceutical management of HF, the prognosis is still poor [3]. Obstructive sleep apneas (OSAs) and central sleep apneas (CSAs) are among the most common comorbidities that are strongly associated with all-cause mortality in more than 50% of patients with HF [4,5]. Several therapeutic approaches have been applied to address CSA and OSA, but none of these pharmacologic interventions have been shown to significantly improve patients’ prognoses for HF [6,7,8,9]. Due to its correlation with poorer left ventricular function and an advanced New York Heart Association (NYHA) class [10], CSA seems to be a significant indication of HF severity, despite OSA being considered an independent risk factor augmenting HF morbidity and death [11,12,13,14,15,16,17], while the benefits of non-invasive ventilation (NIV) for CSA therapy in HF are still debatable [18,19,20], as suggested by the findings of both SERVE-HF [21] and ADVENT-HF [22] randomized clinical trials. Sodium-glucose cotransporter 2 inhibitors (SGLT2i) lower blood glucose levels inhibiting SGLT2 in the renal tubules; however, despite some intriguing hypotheses, the main mechanisms underlying its cardioprotective effects are largely unknown. SGLT2i have been shown to dramatically lower cardiovascular risk in people with type 2 diabetes mellitus (T2DM) and high cardiovascular risk [23,24,25]. In addition, dapagliflozin and empagliflozin have been shown to improve clinical prognosis in patients with HF across the spectrum of ejection fraction regardless of T2DM [26,27,28,29]. Our previous data have demonstrated how therapeutic optimization of Heart Failure with reduced ejection fraction (HFrEF) improves polygraphic parameters, both in OSA and in CSA patients already in treatment with continuous positive airway pressure (CPAP) [30]. In a randomized case–control study conducted on 36 newly diagnosed T2DM and sleep apnea (SA) patients, dapagliflozin showed the potential to be a successful therapy for OSA, due to its effects on lowering blood sugar, body mass index (BMI), and apnea-hypopnea index (AHI), improving hypoxemia during sleep, and reducing excessive daytime drowsiness [31]. Improvement was also shown in both mild and moderate SA [32]. However, the possible role of SGLT2i on improving SA was not demonstrated in HF and T2DM patients not treated with CPAP. Therefore, we postulated that SGLT2i may potentially improve SA patients’ ventilation. Based on these considerations, in order to address this issue in patients with HF, T2DM and SA, considering that medical therapy must be optimized prior to any CPAP therapy [1], our study evaluated the effects of a 3-months treatment with SGLT2i in patients with SA, T2DM, and HF across the spectrum of ejection fraction in patients without CPAP treatment. In this group of patients, we also evaluated the possible predictors of 50% AHI reduction.

## 2. Materials and Methods

### 2.1. Study Population

The Chronic Heart Failure Unit of the Geriatrics Division and Pulmonology Division of the “Renato Dulbecco” University Hospital of Catanzaro made up the study population, which included 514 consecutive outpatients enrolled from October 2020 to September 2023. Elderly outpatients with HF, T2DM, and SA who were eligible for treatment with SGLT2i due to symptom persistence despite good medical therapy were included in the investigation, before starting any CPAP therapy. Written informed consent, age over 65 years, SA occurrence, and no treatment with CPAP were all required. SA diagnosis was performed in accordance with the most recent recommendations [33]. No medication or other substances that could have disrupted sleep were taken by the patient. Acute HF, chronic HF NYHA IV class, severe renal dysfunction (eGFR ≤ 30 mL/min/1.73 m^2^), severe hepatic impairment (Child-Pugh Class C), pregnancy or breastfeeding, relevant valvular heart diseases (VHDs), and treatment with CPAP since at least 3 months were the exclusion criteria. Following these preliminary assessments, two groups were defined considering the patients’ agreement to receive SGLT2i or not: the treated group with SGLT2i, and patients that refuse SGLT2i for own decision. Patients who did not receive SGLT2i continued to receive standard medical care for their co-morbid conditions. All patients were re-evaluated 3 months after the start of therapy for assessment of potential benefits and adverse events.

Each patient had a thorough physical examination, which included evaluations of body mass index (BMI), NYHA functional class, and quality of life. Electrocardiograms (ECGs), echocardiograms, and laboratory tests were performed. After 3 months from the study start, all clinical, laboratory, and instrumental assessments, including echocardiography and nocturnal cardio-respiratory monitoring (CRM), were conducted; in addition, an anamnestic report was used to verify correct adherence to the recommended medical therapy. We considered as the first endpoint a 50% reduction in baseline AHI value.

### 2.2. Polygraphic Parameters

All patients underwent nocturnal CRM (Somtè, Compumedics, Melbourne, Australia). According to the standards of the American Academy of Sleep Medicine, each episode was classified as either obstructive, central and/or mixed, and apnoic and/or hypopnoic by the same operator who was blinded to the treatment regimen [33]. AHI (central/obstructive apnea-hypopnea index) baseline values were used to stratify the patient group and assess the potential existence of SA. Additionally, measurements of oxygen desaturation index (ODI), percentage time of saturation below 90% (TC90), and peripheral arterial oxyhemoglobin saturation (SpO_2_) were made.

### 2.3. Laboratory Parameters

After at least 12 h of fasting, all lab tests were conducted. The glucose oxidase technique (glucose analyzer, BeckmanCoulter, Milan, Italy) was used to detect hyperglycemia. Enzymatic techniques were used to determine the levels of triglycerides, total cholesterol, low-density lipoprotein (LDL) cholesterol, and high-density lipoprotein (HDL) cholesterol in the blood (Roche Diagnostics GmbH, Mannheim, Germany). The Jaffe technique was used to test the levels of creatinine. Based on the updated CKD-EPI (Chronic Kidney Disease Epidemiology Collaboration) equation [34], estimated glomerular filtration rate (eGFR) was assessed. Utilizing the URICASE/POD technique (Boehringer Mannheim, Mannheim, Germany), serum uric acid (UA) levels were measured. Cardio Phase hs-CRP, Milan, Italy, used an automated immunoturbidimetric approach to measure the high sensitivity C-reactive protein (hs-CRP). By using an enzyme-linked immunosorbent test (ElecsysproBNPassay, Roche Diagnostics), the levels of serum N-terminal pro-B-type natriuretic peptide (NT-pro-BNP) were determined.

### 2.4. Echocardiographic Parameters

Echocardiographic recordings were made using a VIVID E-95 ultrasound system (GE Technologies, Milwaukee, WI, USA) equipped with a 2.5 MHz transducer. All patients were examined at rest and in left lateral decubitus. Measurements were obtained according to the recommendations of the American Society of Echocardiography [35,36].

### 2.5. Ethics Committee 

The protocol was approved by the University Ethics Committee (2022.384), and written informed consent was obtained from all participants to the “MAgna GraecIa evaluation of Comorbidities in patients with Heart Failure (MAGIC-HF)” study (ClinicalTrials.gov identifier: NCT05915364) and by the local Ethics Committee of Calabria Region, Italy (Catanzaro, Italy, document n. 263–23 July 2020). This study met the standards of good clinical practice (GCP) and the principles of the Declaration of Helsinki.

### 2.6. Statistical Analysis

Continuous variables are expressed as mean and standard deviation (SD) or as median and interquartile range (IQR) when appropriate. Categorical data are expressed as numbers and percentage, and comparisons between the 2 groups were made with the χ2 test. Comparisons between the 2 groups were made with the *t*-test and Mann–Whitney test for unpaired data when appropriate. In patients that completed the study, changes in polygraphic parameters between baseline and follow-up (ΔT0–3) were compared between patients treated and not treated with SGLT2i using the Mann–Whitney test. To assess the variables linked to the 50% decrease in AHI in patients that completed the study, a logistic regression model was created, with the 50% reduction in AHI serving as the dependent variable and other population variables as covariates. A multivariate stepwise logistic regression model was thus constructed using the variables that substantially linked with the dependent variable to calculate the odds ratio (OR) for the independent predictors associated with the reduction of 50% in AHI. A *p*-value < 0.05 was set for statistical significance. The SPSS V20.0 software for Windows (SPSS Inc., Chicago, IL, USA) was used to conduct the statistical analysis.

## 3. Results

We enrolled 514 elderly patients affected by HF and SA, with a mean age of 76.0 ± 7.1 years. A total of 286 (55.6%) subjects were in treatment with SGLT2i and 228 (44.4%) without SGLT2i: in particular, 172 in treatment with empagliflozin and 114 with dapagliflozin. Of interest, 182 (35.4%) suffered from HF with reduced ejection fraction (HFrEF) and 332 (64.6%) from HF with mildly reduced and preserved ejection fraction. Out of the 512 patients who were enrolled, 398 successfully completed the three-month follow-up. Clinical and laboratory characteristics of enrolled patients, stratified on the basis of SGLT2i therapy, are expressed in Table 1.

The baseline comorbidities and therapies of the complete research group are shown in Table 2.

The baseline echocardiographic and polygraphic features, stratified according to SGLT2i therapy, are included in Table 3.

**Figure 1:**
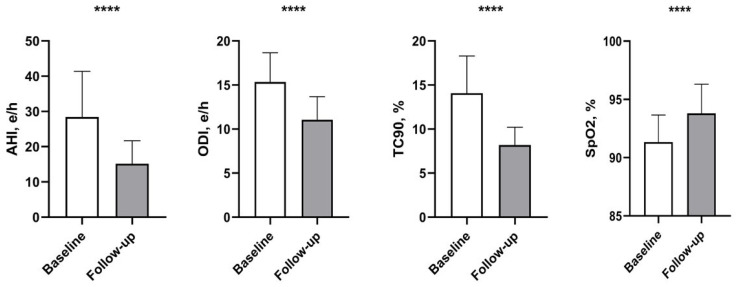
Baseline to follow-up change in polygraphic parameters in patients treated with SGLT2i. Data are mean ± SD. **** *p* < 0.0001 vs. baseline.

In particular, 312 SA patients were characterized by a predominantly central pattern and 202 by a predominantly obstructive pattern. The two groups were similar in terms of the main echocardiographic variables except for global longitudinal strain (GLS), which had worse values in the group not taking SGLT2i. Moreover, other significant differences between the two groups were observed with regard to AHI and TC90 (with worse values in SGLT2i groups) (Table 3). Significant improvements in polygraphic parameters were observed in the entire population when comparing baseline values with 3-month follow-up data. This included a decrease in AHI (27.4 ± 13.3 e/h vs. 15.7 ± 6.4 e/h; *p* < 0.0001), ODI (15.3 ± 3.4 e/h vs. 11.2 ± 2.6 e/h; *p* < 0.0001), and TC90 (13.6 ± 4.1% vs. 7.9 ± 2.0%; *p* < 0.0001). Additionally, there was a substantial increase in mean SpO_2_ (91.3 ± 2.3% vs. 94.0 ± 2.6%; *p* < 0.0001). Values between baseline and follow-up (ΔT0–3) for ΔAHI, ΔODI, ΔTC90, and ΔSpO_2_ were −11 [−24.3/0.01], −4 [−7.00/−1.01], −5 [−9.25/−2.00], and 3 [0.01/5.00], respectively. The study results indicate that the treated group experienced significant improvements in polygraphic parameters between baseline values and follow-up. Specifically, there was a decrease in AHI (28.4 ± 12.9 e/h vs. 15.2 ± 6.5 e/h; *p* < 0.0001), ODI (15.4 ± 3.3 e/h vs. 11.1 ± 2.6 e/h; *p* < 0.0001), and TC90 (14.1 ± 4.2% vs. 8.2 ± 2.0%; *p* < 0.0001), while mean SpO_2_ improved (91. 3 ± 2.3 vs. 93.8 ± 2.5); *p* < 0.0001. (Figure 1); these improvements were not observed in the untreated population. When comparing the Δ of polygraphic parameters between the two groups, some differences were observed, respectively, for ΔAHI (−12 [−26.00/−2.00] vs. −9.5 [−23.00/3.00], *p* < 0.003) and ΔSpO_2_ ([0.01/−4.00] vs. 0.01 [−1.01/5.00], *p* < 0. 011) in the SGLT2i group compared to the non-SGLT2i group, while no differences were observed between the two groups for ΔODI-4 ([−7.00/−2.00] vs. −4 [−7.00/−1.01], *p* < 0.281), ΔTC90 (−6 [−10.00/−2.00] vs. −5 [−9.00/−2.00], *p* < 0.339). Interestingly, in the SGLT2i group, we observed an improvement in right heart function as assessed by tricuspid annular plane systolic excursion (TAPSE) from 18.7 ± 1.9 to 19.5 ± 2.1 mmHg, *p* < 0.0001; we also observed a reduction in systolic pulmonary arterial pressure (s-PAP) from 41.1 ± 5.3 to 38.1 ± 4.6 mmHg, *p* < 0.0001.

Additionally, there was a substantial increase in mean SpO_2_ (91.3 ± 2.3% vs. 94.0 ± 2.6%; *p* < 0.0001) (Figure 1). In the population that completed the study, 220 patients (42.8%) achieved a 50% reduction in AHI during the follow-up period; of these, 86 (49.4%) belonged to group that refused SGLT2i treatment and 134 (59.8%) to the SGLT2i group (*p* = 0.038). Simple logistic regression analysis using the achievement of 50% reduction in AHI as dependent variable revealed that this outcome was significantly associated with ischemic heart disease (IHD) (yes/no) (OR = 0.41; *p* < 0.0001), female gender (yes/no) (OR = 0.51; *p* = 0.004), age (10 years) (OR = 0.65; *p* = 0.029), HOMA-IR (1 pt) (OR = 0.90; *p* = 0.016), inferior vena cava (IVC) (1 mm) (OR = 0.91; *p* = 0.019), GLS (1%) (OR = 0.93; *p* = 0.030), SGLT2i (yes/no) (OR = 1.52; *p* = 0.044), BMI (1 kg/m^2^) (OR = 1.05; *p* = 0.029), and baseline DBP (OR = 1.03; *p* = 0.034) (Table 4).

Moreover, variables significantly correlated to the achievement of 50% reduction in AHI were inserted in a multivariate stepwise logistic regression model to determine the independent predictors of the overmentioned outcome. We identified the following variables as independent predictors of AHI reduction: SGLT2i (yes/no) (OR = 1.6; *p* = 0.004) and BMI (1 kg/m^2^) (OR = 1.05; *p* = 0.022) which were associated with a 50% reduction in the AHI value, while IHD (yes/no) (OR = 0.44; *p* < 0.0001), female gender (yes/no) (OR = 0.51; *p* = 0.002), age (10 years) (OR = 0.61; *p* = 0.023), HOMA-IR (1 pt) (OR = 0.90; *p* = 0.02), and IVC (1 mm) (OR = 0.91; *p* = 0.019) did not increase the odds of achieving 50% reduction in the baseline AHI (Table 5).

## 4. Discussion

The current observational study’s aim was to assess how SGLT2i influenced AHI in patients with HF, T2DM, and SA syndrome. In patients receiving 3 months of SGLT2i treatment without CPAP, we saw substantial improvements in the AHI, ODI, TC90, and mean SpO_2_. Our observational study supports the hypothesis that the therapeutic optimization of HF, in addition to improving its signs and symptoms, also improves sleep-related breathing disorders; in particular, therapy with SGLT2i has been shown to increase 60% the possibility of obtaining a 50% reduction in the AHI value without CPAP treatment. In addition, the reduction of 1 kg/m^2^ in BMI contributed to obtaining an improvement in the baseline AHI value. In contrast, the presence of IHD, female gender, and 10-year augmentation reduced the chance of achieving a 50% reduction in AHI by 56%, 49%, and 39%, respectively. In addition, worsening of insulin resistance (1 pt of HOMA-IR) and worsening of congestion (1 mm change in IVC diameter) reduced the odds of response by 10% and 9%, respectively. The promotion of weight loss is one potential way that SGLT2i might help OSA patients. Diet and weight loss alone may lessen OSA severity. By boosting lipolysis and reducing central adiposity, SGLT2i helps this effect [37]. As fat deposits around the neck and thorax may make upper airway collapsibility worse, losing weight may have a positive impact on airway collapse and AHI. Indeed, the diuretic action of SGLT2i may prevent nocturnal rostral fluid shift and lessen airway collapse while also reducing the severity of SA [38,39,40]. This is of particular interest, because whereas diuretics can reduce the severity of OSA in obese and hypertensive patients by acting through fluid reduction and redistribution, this is not obvious in diabetic patients where resistance to diuretics is often observed [41]. However, in our work, we enrolled patients suffering from HF with mixed sleep breathing disorders, both CSA and OSA, in the absence of CPAP therapy, and suffering from several comorbidities. Furthermore, after three months, we observed a significant improvement in polygraphic parameters. Considering that the use of CPAP in patients with HFrEF is still debated [18,19,20,21,22,23], this study supports the hypothesis that, in patients with HF and SA prior to CPAP therapy, therapeutic optimization with introduction of SGLT2i for treatment of HF results in a clinical and hemodynamic improvement that reduces the need of CPAP therapy and reserves CPAP therapy for patients who do not respond to or do not tolerate optimal medical therapy. In fact, in our work, the introduction of SGLT2i increases the odds of reducing the baseline AHI value by 60%. Despite the therapies available to us and device treatments, SA has a prevalence of around 40% in patients with HF; furthermore, while OSA is a predominantly nocturnal phenomenon, CSA is present at night as well as during the day [1]. The presence of both CSA and OSA increases sympathetic activation and the risk of arrhythmias, leading to worsening of symptoms and reduced survival in HF [3,4,5,6,7]. Despite numerous therapeutic attempts with NIV, so far, no treatment of CSA has shown a prognostic benefit. In particular, two large randomized controlled trials, the CANPAP (continuous positive airway pressure) trial [42] and SERVE-HF (servo-assisted ventilation) [21] showed neutral and negative effects on the survival of patients with CSA and HFrEF, respectively. Therefore, optimization of HF treatment plays a key role in the management of SA patients. According to multiple studies using animal models receiving empagliflozin after myocardial infarction, SGLT2i may also work by inhibiting sympathetic nervous system activation [43]. Through this mechanism, SGLT2i may theoretically lessen circadian sympathetic overactivity and associated symptoms in OSA patients, including nocturnal hypertension, the non-dipping phenomena [44]. Dapagliflozin only significantly improves moderate-to-severe sleep disordered breathing (SDB), not mild SDB, according to Furukawa et al. [32]. This was the first trial to evaluate dapagliflozin’s effectiveness for SDB in Japanese patients with obesity and T2DM, according to the paper. It had a number of flaws. First off, the study only included 30 patients, making more research with larger sample numbers necessary to validate its conclusions. Second, there was no control group in this research. The results of dapagliflozin therapy were contrasted with the initial characteristics. Thirdly, a home pulse oximeter was used to evaluate SDB rather than polysomnography. T2DM patients with OSA who received SGLT2i and had their AHI evaluated at least twice, both before and after the administration of SGLT2i at a consistent dose, were included in the study by Sawada et al. [45]. The majority of these individuals were obese and had severe OSA. This research discovered that individuals with severe OSA had less improvement in AHI with higher weight reduction. In order to evaluate the impact of adding dapagliflozin to the anti-diabetes regimen in T2DM and OSA patients, Tang et al. performed case–control research [31]. The dapagliflozin-treated group had a decrease in AHI, highlighting the medication’s advantages over an OSA patient’s ability to breathe on their own. OSA patients exhibited a decreased incidence of MACE, mortality, and renal outcomes after using empagliflozin. The underlying mechanisms of such protective benefits include a reduction in oxidative stress, inflammation activation, sympathetic activation, and chronic intermittent hypoxia. A well-known effect of SGLT2i is weight loss, which is a crucial part of managing OSA. Those with OSA may lose more weight with SGLT2 inhibition than those without OSA, according to the secondary analysis of EMPA-REG OUTCOME by Neeland and colleagues [46]. Subcutaneous and visceral adipose tissue decrease as a consequence of SGLT2-inhibitor-related weight loss. In particular, by redistributing fluid without significantly lowering BMI, SGLT2i may help to improve AHI values. Our work has several strengths, such as the large sample size, having enrolled elderly patients with several comorbidities who are often underrepresented in clinical trials, the control group, and suggests that in patients with HF and SA, SGLT2i is an excellent treatment option. However, our investigation has some limitations: it is not a randomized controlled clinical trial so a selection bias cannot be excluded, and we used a CRM and not polysomnography with electroencephalographic channels, which could better characterize the sleep pattern. Finally, we cannot exclude night-to-night AHI variability. Future research is needed to determine how SGLT2i affects SA. In order to compare SGLT2i to presently available noninvasive treatments, such as CPAP and BiPAP, in diabetic and non-diabetic individuals with SA separately, further research is required. To further investigate the efficacy and limitations of SGLT2 inhibition, randomized clinical studies, including the objective measurement of SA by polysomnography, are required.

## 5. Conclusions

In conclusion, our research suggests that the use of SGLT2i in patients suffering from mixed-type sleep-disordered breathing not on CPAP therapy, T2DM, and HF across the spectrum of EF significantly contributes to improving polygraphic parameters. In particular, the use of SGLT2i increases the probability of obtaining a halving of the AHI value by 60%.

## Figures and Tables

**Table 1:** Clinical, anthropometric, and laboratoristic characteristic of the study population at baseline.

	All Population (n. 514)	Without SGLT2i (n. 228)	With SGLT2i (n. 286)	*p*
Age, years	76.3 ± 7.1	76.4 ± 6.8	76.2 ± 7.2	0.702
MLHFQ, pt	83.8 ± 3.7	83.9 ± 3.7	83.7 ± 7.7	0.509
BMI, Kg/m^2^	31.6 ± 4.8	31.3 ± 3.9	31.8 ± 5.5	0.177
SBP, mmHg	121.9 ± 14.5	120.2 ± 14.7	123.3 ± 14.2	0.017
DBP, mmHg	72.3 ± 9.0	71.0 ± 9.2	73.4 ± 8.8	0.004
HR, bpm	68.3 ± 12.2	68.8 ± 9.9	67.8 ± 13.7	0.372
RR, afm	16.3 ± 1.8	16.3 ± 1.8	16.3 ± 1.7	0.864
eGFR, mL/min/1.73 m^2^	65.8 ± 15.7	67.7 ± 17.1	64.4 ± 14.3	0.018
Uric acid, mg/dL	6.7 ± 0.4	6.7 ± 0.4	6.6 ± 0.4	0.587
Hb, g/dL	11.8 ± 1.9	11.6 ± 1.8	12.0 ± 1.9	0.018
HTC, %	33.5 ± 6.6	32.8 ± 6.5	34.0 ± 6.6	0.032
Na^+^, mmmol/L	140.1 ± 3.4	140.1 ± 3.9	140.2 ± 3.0	0.721
K^+^, mmol/L	4.6 ± 0.4	4.6 ± 0.4	4.6 ± 0.4	0.821
NTpro-BNP, pg/mL	1539.8 ± 647.7	1558.1 ± 732.5	1525.2 ± 572.1	0.567
HOMA-IR, pt	8.8 ± 2.5	8.4 ± 2.0	9.1 ± 2.8	0.004
HbA1c, %	7.5 ± 0.5	7.5 ± 0.4	7.5 ± 0.5	0.726
hs-CRP, mg/L	5.3 ± 0.3	5.3 ± 0.3	5.3 ± 0.3	0.205

Abbreviations: MLHFQ: Minnesota living with heart failure questionnaire, BMI: Body mass index, SBP: Systolic blood pressure, DBP: Diastolic blood pressure, HR: heart rate, RR: respiratory rate, e-GFR: estimate glomerular filtration rate, HTC: hematocrit, Hb: haemoglobin, Na: Sodium, K: potassium, NTpro-BNP: N-terminal pro-B-type Natriuretic Peptide, HOMA-IR: Homeostasis Model Assessment Insulin Resistance, HbA1c: glycated haemoglobin, hs-CRP: high sensitive C-reactive protein.

**Table 2:** Comorbidities and therapies of the study population at baseline.

	All Population (n. 514)	Without SGLT2i (n. 228)	With SGLT2i (n. 286)	*p*
IHD, *n* (%)	320 (62.3)	138 (60.5)	182 (63.6)	0.469
Atrial Fibrillation, *n* (%)	156 (30.4)	68 (29.8)	88 (30.8)	0.816
HFrEF, *n* (%)	182 (35.4)	90 (39.5)	92 (32.2)	0.085
HFmrEF-HFpEF, *n* (%)	332 (64.6)	138 (60.5)	194 (67.8)	0.085
Arterial Hypertension, *n* (%)	360 (70)	154 (67.5)	206 (72.0)	0.270
COPD, *n* (%)	136 (26.5)	58 (25.4)	78 (27.3)	0.639
Dislipidemia, *n* (%)	294 (57.2)	124 (54.3)	170 (59.4)	0.249
CKD, *n* (%)	166 (32.3)	72 (31.6)	94 (32.9)	0.756
ICD-CRTd, *n* (%)	312 (60.7)	144 (63.1)	168 (58.7)	0.308
ACEi/ARBs, *n* (%)	204 (39.7)	80 (35.0)	124 (43.4)	0.056
Sacubitril-Valsartan, *n* (%)	182 (35.4)	90 (39.5)	92 (32.2)	0.085
Loop-Diuretics, *n* (%)	450 (87.5)	192 (84.2)	258 (90.2)	0.040
MRAs, *n* (%)	260 (50.6)	116 (50.1)	144 (50.3)	0.905
β-blockers, *n* (%)	352 (68.5)	166 (72.8)	186 (65.0)	0.059
OAC, *n* (%)	156 (30.4)	68 (29.8)	88 (30.8)	0.816
Antiplatelet, *n* (%)	316 (61.5)	148 (64.9)	168 (58.7)	0.153
Statins, *n* (%)	342 (66.5)	164 (71.9)	178 (62.2)	0.020

Abbreviations: IHD: Ischemic heart disease; HFrEF: Heart Failure with reduced ejection fraction; HFmrEF: Heart Failure with mildly reduced ejection fraction; HFpEF: Heart Failure with preserved ejection fraction; COPD: Chronic Obstructive Polmunary Diseas; Broncopneumopatia cronica ostruttiva; ICD-CRTd; CKD: Chronic Kidney Diseas; ACEi: Angiotensin-converting enzyme inhibitors; ARBs: Angiotensin II receptor blockers; MRAs: mineral receptor antagonists; OAC: oral anticoagulant; SGLT2i: Sodium-glucose cotransporter type 2 inhibitor.

**Table 3:** Echocardiographic and polygraphic characteristics of thestudy population at baseline.

	All Population (n. 514)	Without SGLT2i (n. 228)	With SGLT2i (n. 286)	*p*
LAVi, mL/m^2^	46.5 ± 10.5	46.8 ± 10.1	46.2 ± 10.8	0.571
LVEDV/BSA, mL/m^2^	87.3 ± 7	87.7 ± 6.6	86.9 ± 7.3	0.237
LVESV/BSA, mL/m^2^	52.3 ± 6.2	52.8 ± 5.9	51.8 ± 6.4	0.090
LVEF, %	40.2 ± 4.6	39.8 ± 4.6	40.4 ± 4.6	0.126
Cardiac Index, L/min/m^2^	1978.4 ± 193.6	1963.9 ± 194.3	1990.1 ± 192.7	0.128
E/A	0.83 ± 0.4	0.81 ± 0.4	0.85 ± 0.5	0.241
E/e’, pt	17.2 ± 3.5	17.5 ± 3.6	17.0 ± 3.5	0.092
GLS, %	−11.3 ± 3.6	−10.9 ± 4.1	−11.6 ± 3.2	0.031
RVOTp, cm	2.5 ± 0.5	2.5 ± 0.4	2.5 ± 0.5	0.747
RAA, cm^2^	18.9 ± 4.5	18.9 ± 3.8	19.0 ± 4.9	0.812
TAPSE, mm	18.5 ± 1.8	18.4 ± 1.8	18.7 ± 1.9	0.047
s-PAP, mmHg	41.0 ± 5.0	41.0 ± 4.6	41.1 ± 5.3	0.947
TAPSE/s-PAP, mm/mmHg	0.46 ± 0.07	0.45 ± 0.07	0.46 ± 0.06	0.262
IVC, mm	19.4 ± 2.6	19.3 ± 2.3	19.5 ± 2.8	0.375
AHI, n/h	27.4 ± 13.3	26.0 ± 13.6	28.4 ± 12.9	0.041
ODI, n/h	15.3 ± 3.4	15.1 ± 3.4	15.4 ± 3.3	0.484
SpO_2_, %	91.3 ± 2.3	91.2 ± 2.2	91.3 ± 2.3	0.537
TC90, %	13.6 ± 4.1	13.1 ± 4.0	14.1 ± 4.2	0.009

Abbreviations: LAVi: left atrial volume index; LVEDV/BSA: left ventricular end-diastolic volume index/body surface area; LVESV/BSA: left ventricular end-systolic volume index/body surface area; LVEF: left ventricular ejection fraction; E/A: ratio between wave E (the wave of rapid filling in early diastole) and wave A (the wave of atrial contraction); E/e’: between wave E and wave e’ (reliable estimate of changes in end-diastolic blood pressure); GLS: global longitudinal strain; RVOTp: Right Ventricular Outflow Tract proximal; RAA: Right Atrium Area; TAPSE: Tricuspid annular plane systolic excursion; s-PAP: systolic pulmonary arterial pressure; IVC: inferior vena cava; AHI: apnea hypopnea index; ODI: oxygen desaturation index; SpO_2_: peripheral arterial oxyhemoglobin saturation; TC90: percentage time of saturation below 90%.

**Table 4:** Univariate logistic regression focused on the 50% reduction in the AHI value.

	OR	CI 95%	*p*
IHD, yes/no	0.41	0.26–0.63	<0.0001
Female gender, yes/no	0.51	0.32–0.80	0.004
HbA1c, 1%	0.76	0.50–1.18	0.226
Uric acid, 1 mg/dL	0.77	0.47–1.26	0.297
Sac/Val, yes/no	0.82	0.29–2.25	0.703
Arterial Hypertension, yes/no	0.83	0.53–1.31	0.433
E/A	0.85	0.52–1.39	0.522
Hb, 1 g/dL	0.88	0.68–1.12	0.374
HOMA-IR, 1 pt	0.90	0.83–0.98	0.016
IVC, 1 mm	0.91	0.82–0.98	0.019
GLS, 1%	0.93	0.87–0.99	0.030
β-blockers, yes/no	0.95	0.57–1.56	0.827
E/e’, 1 pt	0.98	0.92–1.05	0.569
Loop Diuretics, yes/no	0.98	0.52–1.84	0.955
SGLT2i, yes/no	1.52	1.01–2.29	0.044
Age, 10 years	0.65	0.35–0.92	0.029
CKD, yes/no	1.20	0.75–1.91	0.445
ACEi/ARB, yes/no	1.19	0.69–2.08	0.525
BMI, 1 Kg/m^2^	1.05	1.01–1.10	0.029
HCT, 1%	1.05	0.96–1.15	0.282
DBP, 1 mmHg	1.03	1.01–1.05	0.034
LVEDV/BSA, 1 mL/m^2^	1.03	0.99–1.08	0.130
HR, 1 bfm	1.01	0.99–1.02	0.171
SBP, 1 mmHg	1.00	0.98–1.01	1.000

Abbreviation: IHD: Ischemic heart disease; HbA1c: glycated haemoglobin; Sac-Val: Sacubitril-Valsartan; E/A: ratio between wave E (the wave of rapid filling in early diastole) and wave A (the wave of atrial contraction); Hb: haemoglobin; HOMA-IR: Homeostasis Model Assessment Insulin Resistence; IVC: inferior vena cava; GLS: global longitudinal strain; E/e’: between wave E and wave e’ (reliable estimate of changes in end-diastolic blood pressure); SGLT2i: Sodium-glucose cotransporter type 2 inhibitor; CKD: Chronic Kidney Diseas; ACEi: Angiotensin-converting enzyme inhibitors; ARB: Angiotensin II receptor blocker; BMI: Body mass index; HTC: hematocrit; HR: heart rate; SBP: Systolic blood pressure; LVEDV/BSA: left ventricular end-diastolic volume index/body surface area.

**Table 5:** Multivariate stepwise logistic regression model focused on the 50% reduction in the AHI value.

	OR	CI 95%	*p*
SGLT2i, yes/no	1.60	1.08–2.35	0.004
BMI, 1 kg/m^2^	1.05	1.01–1.09	0.022
IHD, yes/no	0.44	0.29–0.67	<0.0001
Female gender, yes/no	0.51	0.34–0.78	0.002
Age, 10 years	0.61	0.32–0.85	0.023
HOMA-IR, 1 pt	0.90	0.83–0.98	0.020
IVC, 1 mm	0.91	0.84–0.98	0.019

Abbreviation: SGLT2i: Sodium-glucose cotransporter type 2 inhibitor; BMI: Body mass index; IHD: Ischemic heart disease; HOMA-IR: Homeostasis Model Assessment Insulin Resistance; IVC: inferior vena cava.

## Data Availability

The raw data supporting the conclusions of this article will be made available by the authors, without undue reservation.

## Abbreviations

ACEi	angiotensin-converting enzyme inhibitors
AHI	apnea hypopnea index
ARBs	Angiotensin II receptor blockers
BMI	body mass index
CKD	Chronic Kidney Disease
COPD	Chronic Obstructive Pulmonary Disease; Broncopneumopatia cronica ostruttiva
CPAP	Continuous Positive Air Pressure
CSA	Central Sleep Apneas
DBP	diastolic blood pressure
E/A	ratio between wave E (the wave of rapid filling in early diastole) and wave A (the wave of atrial contraction)
E/e’	ratio between wave E and wave e’ (reliable estimate of changes in end-diastolic blood pressure)
GLS	global longitudinal strain
e-GFR	estimate glomerular filtration rate
Hb	Haemoglobin
HbA1c	glycated haemoglobin
HF	Heart Failure
HFrEF	Heart Failure with reduced ejection fraction
HFmrEF	Heart Failure with mildly reduced ejection fraction
HFpEF	Heart Failure with preserved ejection fraction
HOMA-IR	Homeostasis Model Assessment Insulin Resistance
HR	heart rate
hs-CRP	high sensitive C-reactive protein
HTC	Hematocrit
IHD	ischemic heart disease
IVC	inferior vena cava
K	Potassium
LAVi	left atrial volume index
LVEDV/BSA	left ventricular end-diastolic volume index/body surface area
LVEF	left ventricular ejection fraction
LVESV/BSA	left ventricular end-systolic volume index/body surface area
MLHFQ	Minnesota living with heart failure questionnaire
MRAs	mineral receptor antagonists
Na	Sodium
NIV	Non Invasive Ventilation
NTpro-BNP	N-terminal pro-B-type Natriuretic Peptide
OAC	oral anticoagulant
ODI	oxygen desaturation index
OSA	obstructive sleep apneas
RAA	Right Atrium Area
RR	respiratory rate
RVOTp	Right Ventricular Outflow Tract proximal
SA	Sleep Apnea
SBP	systolic blood pressure
SGLT2i	sodium-glucose cotransporter type 2 inhibitor
s-PAP	systolic pulmonary arterial pressure
SpO_2_	peripheral arterial oxyhemoglobin saturation
T2DM	type 2 diabetes mellitus
TAPSE	tricuspid annular plane systolic excursion
TC90	percentage time of saturation below 90%
VHD	valvular heart disease
